# Molecular Testing of Zoonotic Bacteria in Cattle, Sheep, and Goat Abortion Cases in Botswana

**DOI:** 10.3390/microorganisms12122644

**Published:** 2024-12-20

**Authors:** Boitumelo M. Modise-Tlotleng, Sununguko W. Mpoloka, Tirumala B. K. Settypalli, Joseph Hyera, Tebogo Kgotlele, Kago Kumile, Mosarwa E. Sechele, Obuile O. Raboloko, Chandapiwa Marobela-Raborokgwe, Gerrit J. Viljoen, Giovanni Cattoli, Charles E. Lamien

**Affiliations:** 1 National Agricultural Research and Development Institute, Private Bag 0035, Gaborone, Botswana; boikensmod@gmail.com (B.M.M.-T.); tkgotlele@nardi.org.bw (T.K.); kkumile@nardi.org.bw (K.K.); 2 Department of Biological Sciences, University of Botswana, Private Bag 00704, Gaborone, Botswana; mpoloka@ub.ac.bw; 3 Animal Production and Health Laboratory, Joint FAO/IAEA Centre of Nuclear Techniques in Food and Agriculture, Department of Nuclear Sciences and Applications, International Atomic Energy Agency, Wagramer Strasse 5, P.O. Box 100, 1400 Vienna, Austria; t.b.k.settypalli@iaea.org (T.B.K.S.); g.j.viljoen@gmail.com (G.J.V.); 4 Botswana Vaccine Institute, Private Bag 0031, Gaborone, Botswana; jhyera@bvi.co.bw; 5 Department of Veterinary Services (DVS), Ministry of Agriculture, Private Bag 0032, Gaborone, Botswana; mesechele@gmail.com (M.E.S.); ooraboloko@gmail.com (O.O.R.); chandapiwamarobela@gmail.com (C.M.-R.); 6 Istituto Zooprofilattico Sperimentale delle Venezie, Viale dell’Università 10, 35020 Legnaro, Italy; gcattoli@izsvenezie.it

**Keywords:** brucellosis, leptospirosis, coxiellosis, listeriosis, syndromic surveillance, zoonotic

## Abstract

Abortion is one of the major causes of economic losses in livestock production worldwide. Because several factors can lead to abortion in cattle, sheep and goats, laboratory diagnosis, including the molecular detection of pathogens causing abortion, is often necessary. Bacterial zoonotic diseases such as brucellosis, coxiellosis, leptospirosis, and listeriosis have been implicated in livestock abortion, but they are under diagnosed and under-reported in most developing countries, including Botswana. This study applied a recently developed multiplex high-resolution melting analysis technique, coupled with singleplex qPCR assays, to investigate abortions in livestock in Botswana, using 152 samples from cattle, sheep, and goat abortion cases. *Brucella* spp. were the most frequent pathogen detected, with an overall frequency of 21.1%, followed by *Coxiella burnetii* with 19.1%. *Listeria monocytogenes* and *Leptospira* spp. were not detected in any of specimens samples investigated. Mixed infections with *Brucella* spp. and *C. burnetii* were observed in 35% specimes examined. There was a good agreement between the multiplex qPCR-HRM and singleplex qPCR for detecting *Brucella* spp. and *C. burnetii*. This study is the first report on the syndromic testing of abortion-causing pathogens in Botswana. It shows the importance of molecular methods in the differential diagnosis of abortion-causing diseases in domestic ruminants.

## 1. Introduction

Livestock farming plays a significant role in the livelihoods of most Batswana by providing food, income, employment, and cultural attributes for most rural communities. Botswana’s livestock production comprises communal/traditional and commercial land tenure systems [1,2]. Communal farming occurs on communal or tribal lands with open access to rangeland, leading to uncontrolled grazing, minimal fencing, and a reliance on borehole-centered cattle posts. It accounts for 86% of the national cattle herd, and 71% of Botswana farmers use open-access grazing lands for their herds [1,2,3]. In contrast, commercial farming involves fenced grazing areas on freehold or leasehold ranches, promoting managed production [1,2]. It accounts for 14% of the national cattle herd and 5% of the land area [1,2,3]. In Botswana, the holdings and population of cattle, sheep, and goats stand at approximately 29,355 holdings with 935,000 cattle; 42,179 holdings with 1,229,000 sheep; and 11,439 holdings with 242,911 goats (Table S1) [4].

Livestock abortions result in significant economic losses due to decreased productivity, loss of breeding stock, and potential trade impacts. Infectious agents are the main cause of abortion in livestock as compared to non-infectious agents [5,6]. The specific percentage of cases caused by infectious agents remains uncertain. However, in 90% of cases where an etiological diagnosis is determined, the cause is infectious [7,8]. This includes pathogens such as bacteria, viruses, protozoa, and fungi, which are frequent causes of abortions in ruminants. Bacterial pathogens, such as *Brucella* spp., *C. burnetii*, *Leptospira* spp., and *L. monocytogenes*, are common infectious agents that cause abortions in farmed ruminants and are of zoonotic importance [9]. These bacterial pathogens lead to animal diseases (brucellosis, coxiellosis, leptospirosis, and listeriosis) that significantly threaten the livestock industry and cause substantial economic losses [10,11,12]. These diseases not only impact animal health and productivity, but also affect veterinary public health, and trade in livestock and livestock products. The economic impact extends to livestock farmers, hindering rural income generation and job creation, and most importantly, compromising food security [10,13]. Many of these pathogens can also spread to humans, raising animal and public health concerns.

Brucellosis, caused by bacteria of the genus *Brucella*, is a significant worldwide infectious disease of domestic and wild animals with humans as incidental hosts [14,15]. This disease is widespread across Africa, particularly in the sub-Saharan region, with varying prevalence depending on the geographical location. In cattle, prevalence ranges from 1.0% to 36.6%, in goats from 0.0% to 5.5%, in sheep from 0.0% to 4.8%, and in humans from 0.15% to 24.1% [16]. In animals, the disease is characterized by abortion during the middle to last trimester, retained placenta, stillbirth, low milk production, epididymitis, orchitis, infertility, and fetal death [17,18]. Animals contract the infection by consuming contaminated milk, feed, or water, by contacting aborted materials, infected animals, semen, and uterine discharge or by inhaling aerosols from different sources [19,20,21]. Infection can also be spread through mating and artificial insemination [22,23].

Coxiellosis is a zoonotic disease caused by *C. burnetii*, a highly infectious bacterium classified in the genus *Coxiella* [24]. This disease is globally distributed, except in Antarctica and New Zealand, and is likely widespread in Africa, although it remains underreported [25,26,27]. The host reservoirs include mammals, birds, and arthropods, but domestic ruminants such as cattle, sheep, and goats are typically major reservoirs [28]. Coxiellosis is often asymptomatic or subclinical in ruminants, but late abortions, stillbirths, and reproductive disorders occur occasionally [29,30]. *C. burnetii* can induce epidemics of reproductive failure in sheep and goats but not in cattle [31]. Infection with *Coxiella* may occur through inhalation of contaminated aerosols, ingestion of contaminated feed, tick bites, and semen [32,33,34].

Leptospirosis is a globally significant bacterial infection of animals and humans with ubiquitous distribution, caused by pathogenic spirochetes in the genus *Leptospira*. This disease is endemic in sub-Saharan Africa [35]. Pathogenic *Leptospira* has reservoirs in many animals, including rodents and livestock [36]. Acute and chronic infections are more common in cattle than in sheep and goats, often presenting subclinically, especially in non-pregnant and non-lactating animals [37]. Acute infections in calves can be severe, with symptoms including high fever, hemolytic anemia, hemoglobinuria, jaundice, pulmonary congestion, and potentially meningitis or death. Lactating cows experience a significant drop in milk production. Chronic infections can lead to abortions, often accompanied by a retained placenta or weak or stillborn calves [37]. *Leptospira* infections in animals occur through direct contact with urine or indirectly through contaminated materials such as water, fodder, grass, saliva, semen, milk, postpartum tissues, and vectors (flies and mosquitoes) [38,39,40]. *Leptospira* can also be transmitted venereally, and vertically through the placenta.

Listeriosis is a fatal infectious bacterial disease caused by *L. monocytogenes*, of the genus *Listeria* [41,42]. While it occurs globally, it is more commonly found in temperate and colder climates [43]. It affects a wide variety of animal species including mammals, birds, fish, and crustaceans [44]. The most susceptible domestic species are sheep, goats, and cattle [45]. Listeriosis causes encephalitis, abortion, mastitis, repeat breeding, and endometriosis in animals [46]. Abortion storms are more common in sheep and most abortions occur after the 12th week of pregnancy. In cattle, abortions are sporadic and occur within the last trimester [44,47]. Infected animals shed *L. monocytogenes* in the faeces, milk, and uterine discharges and can be detected in soil, vegetables, sewage, drains, bedding, water and food, animals, and the human intestine [48,49,50]. *Listeria* infections are acquired through ingesting contaminated feed and water, inhalation, or direct contact [44,51], and venereal transmission [47].

Accurate laboratory diagnostics, including rapid molecular tests, are essential for early detection, identifying the specific causative agent, and enabling effective control measures. This study aimed to identify abortion-causing bacteria—*Brucella* spp., *Leptospira* spp., *C. burnetii*, and *L. monocytogenes*—in samples from cattle, goats, and sheep in Botswana using a novel multiplex real-time polymerase chain reaction (qPCR) method based on high-resolution melting (HRM) curve analysis, coupled with singleplex qPCR tests.

## 2. Materials and Methods

### 2.1. Ethics Approval and Participation Consent

The present study followed international ethical guidelines and was evaluated and approved by the Animal Care and Use Committee of the Office of Research and Development, University of Botswana (UBR/RES/ACUC/016). The Ministry of Agriculture (MOA) provided permission to conduct the study and test samples from cattle, sheep, and goats, with Reference No: MOA 1/15/4 II (6). Samples used were provided by Botswana National Veterinary Laboratory (BNVL, previously tested for routine diagnosis). The authors confirm that this study was conducted following ARRIVE guidelines [52].

### 2.2. Study Areas and Sample Collection

Syndromic testing was performed using 152 clinical samples, available at the time of testing, from cattle, sheep, and goats originating from all ten different districts of Botswana (Chobe, North-West (Ngamiland), North-East, Central, Ghanzi, Kgalagadi, Southern, Kweneng, Kgatleng, and South-East) (Tables S1 and S2). The samples were submitted by field personnel to BNVL for routine testing and comprised archived abortion cases from 2010 to 2019 as well as direct clinical outbreaks or sporadic cases from 2019 to 2021. Epidemiological information including case history, animal species, and geographical location was collected from original sample submission forms that accompanied the samples submitted to BNVL (Table S2). Specimens consisted of livers, spleens, ovary tubes, vaginal swabs, kidneys, bladders, lymph nodes, brains, placenta, abdominal/stomach contents, whole blood, and sera.

In addition, opportunistic testing was performed on milk samples (34) from cattle from the BNVL Dairy Hygiene Unit used for routine quality control testing. The multiplex qPCR-HRM assay results for 186 samples (152 abortive and 34 milk samples) were confirmed with each of the four singleplex qPCRs (*Brucella* spp., *L. monocytogenes*, *C. burnetii*, and *Leptospira* spp.).

### 2.3. Detection of Nucleic Acids

Nucleic acid (DNA) was extracted from the samples using the DNeasy Blood & Tissue Kit (Qiagen, Hilden, Germany) as per the manufacturer’s instructions with some modifications [53]. These modifications are that instead of adding animal tissue lysis (ATL) buffer from the kit the grinding of tissue and incubating with Proteinase K at 56 °C, the homogenized tissue supernatants or cell culture supernatants were prepared by adding Qiagen RLT plus lysis buffer (200 μL sample + 800 μL RLT plus buffer). The extracted DNA was tested using a recently developed novel multiplex qPCR-HRM assay for the simultaneous detection and differentiation of four abortive zoonotic agents in cattle, sheep, and goats [54]. This assay is based on a high-resolution melting curve analysis of PCR amplicons produced using specific primer pairs and double-stranded DNA-binding dye, and it exploits the differences in fragment size and GC content for discrimination. The method generates four well-separated melting peaks between the four zoonotic abortifacients.

Briefly, a 20 μL PCR reaction volume containing 1 × SsoFast™ EvaGreen^®^ Supermix (BioRad, Hercules, CA, USA), 150 nM of each primer pair of *Brucella* spp., *L. monocytogenes* and *C. burnetii*, and 350 nM of *Leptospira* spp., and 2 μL of DNA template was prepared. The PCR was carried out using the CFX96 Touch Real-Time PCR Detection System (Bio-Rad, USA) with the following cycling conditions: initial denaturation at 95 °C for 5 min, 42 cycles with denaturation at 95 °C for 5 s, annealing at 62 °C for 4 s, and an extension at 70 °C for 5 s. Following the completion of PCR, products were subjected to the following melting program: denaturation at 95 °C for 1 min, cooling to 65 °C for 1 min and with continuous heating at 0.2 °C increments every 10 s and with fluorescence acquisition from 65 °C to 90 °C. Each PCR run included a positive control for each of the four bacteria, as previously described [54], a no-template control (NTC) consisting of water, and a negative extraction control, which consisted of water subjected to the full extraction process. The following melting temperature (Tm) value ranges were used to identify the bacteria: 83.0 ± 0.6 °C for *Brucella* spp., 80.4 ± 0.6 °C for *C. burnetii*, 77.4 ± 0.4 °C for *L. monocytogenes*, and 75.5 ± 0.5 °C for *Leptospira* spp.

The samples were also subjected to detection using probe-based (Taqman) and DNA-intercalating fluorescent dye assays. The Taqman PCR assays were for the detection of *Brucella* spp., targeting the *IS711* gene [9], the detection of *C. burnetii*, targeting the *IS1111* gene [55], and the detection of *Leptospira* spp., targeting the *lipL32* gene [56] while the DNA-intercalating fluorescent dye assay for *L. monocytogenes* targeted the *ssrA* gene [57]. The probe-based qPCR amplifications were carried out by combining 1 × iQ Supermix, primers (Table S3), a 5′FAM^®^-labeled probe with a 3′BHQ1 quencher dye (Table S3), and 2 µL of the DNA template, amounting to a final volume of 20 µL using nuclease-free water. The PCR cycling conditions used are provided in Table S4. The *L. monocytogenes* detection assay was performed with some modifications using primers (Table S3), 1 × SsoFast™ EvaGreen^®^ Supermix (BioRad, Hercules, CA, USA), and 2 µL of the DNA in a final reaction volume of 20 µL, adjusted with nuclease-free water. The assay was conducted under the following cycling conditions: an initial denaturation step at 95 °C for 5 min, followed by 45 cycles of 95 °C for 15 s and 60 °C for 60 s with fluorescence acquisition performed at the end of each cycle. The PCR products were then denatured at 95 °C (held for 60 s), cooled to 65 °C (held for 60 s), and melted from 65 °C to 90 °C with a 0.5 °C temperature increment every 0.05 s with continuous data acquisition. A negative extraction control, NTC, and positive control were included in each run.

### 2.4. Data Analysis and Statistical Analysis

The generated data were entered into WPS Office Spreadsheet 2016 and analyzed using Orange 3.34.0 data mining software and Microsoft Excel to determine the most frequent pathogen among the four abortion-causing agents, detect mixed infections, and evaluate the distribution of the pathogens between districts. In addition, the agreement between the newly developed multiplex qPCR-HRM assay for the simultaneous detection of four abortive zoonotic agents in domestic ruminants and the existing singleplex qPCR assays for the same pathogens was evaluated using Cohen’s kappa coefficient and Bland-Altman analysis. Cohen’s kappa coefficient (κ) is a statistic that assesses inter-rater reliability between observers or measurements of the same categorical variable. Kappa values between 0.80 and 1 are considered perfect agreement [58]. Bland-Altman analysis is a simple way to evaluate a bias between the mean differences and estimate the agreement interval, within which 95% of the differences between the second and the first methods fall [59].

## 3. Results

The case history from syndromic surveillance indicated that all the affected animals displayed symptoms of abortion at various stages of gestation (first, second, and third). Of the 152 samples, 115 (75.7%) tested positive for abortion-causing pathogens using the multiplex qPCR-HRM assay. The pathogens were detected in a range of sample matrices, mostly in the stomach contents and liver/spleen from foetuses (Table 1). Of the 115 positive samples, *Brucella* spp. were detected in 32 samples (27.8%), *C. burnetii* in 29 samples (25.2%), and mixed infections with *Brucella* spp. and *C. burnetii* in 54 samples (47.0%) (Table 2). *Brucella* spp. were the most frequently detected pathogens with frequency of 21.1%, (32/152), followed by *C. burnetii* with a frequency of 19.1% (29/152). Analysis per animal species showed that *C. burnetii* was the most detected pathogen in goats (21.4%; 24/112), while *Brucella* spp. were mostly detected in cattle (34.6%; 9/26), and there was an equal positive number of *C. burnetii* and *Brucella* spp. in sheep (21.4%; 3/14; Table 2). In addition, mixed infections involving *C. burnetii* and *Brucella* spp. were detected in cattle, sheep, and goats (Table 2). Of the 54 mixed infections, a high number of 37 (68.5%) was detected in goats, followed by 11 (20.4%) in cattle and 6 (11.1%) in sheep.

Opportunistic testing of milk samples detected *Brucella* spp. (4), *C. burnetii* (9), and the co-occurrence of *Brucella* spp. and *C. burnetii* (4) using the newly developed multiplex qPCR-HRM assay.

Of the 186 samples (syndromic and opportunistic samples), the multiplex qPCR-HRM and *Brucella* spp. Taqman qPCR assays detected 94 *Brucella* spp. and 92 negative samples while the multiplex qPCR-HRM and *C. burnetii* Taqman PCR assays detected 96 *C. burnetii* and 90 negative samples. A perfect agreement with kappa = 1 was observed when comparing the positive and negative results of the multiplex qPCR-HRM assay and each of the singleplex qPCRs. Similarly, the Bland-Altman analysis for HRM and *Brucella* spp. qPCR showed a mean Cq difference of −0.7 with 95% limits of agreement ranging from −7.464 to 5.989 (Figure S1). The mean Cq difference between HRM and *C. burnetii* qPCR was 0.63, with 95% confidence limits of agreement ranging from −4.244 to 5.504 (Figure S2). Both analyses suggested a good agreement between the two methods. The samples were negative for *Leptospira* spp. and *L. monocytogenes* using both the multiplex HRM and singleplex qPCR assays.

**Figure 1:**
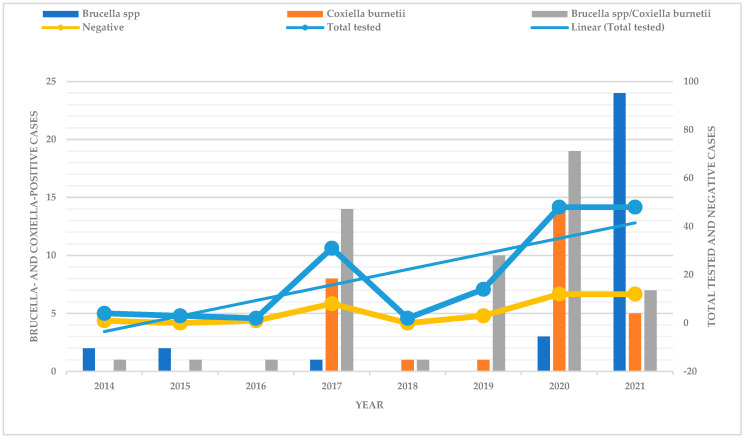
Distribution of *Brucella-* and *Coxiella*-positive cases from 2014 to 2021.

*Brucella*-positive cases were low in 2014, 2015, and 2017 and there were no positive cases in 2016, 2018 and 2019. An increase was observed in 2020, and a significant increase in 2021 (Figure 1). There were no *C. burnetii*-positive cases observed in 2014, 2015 and 2016, a low number of positive cases were recorded in 2018 and 2019, and an increase occurred in 2017 and 2021, with the highest number of positive cases in 2020. Mixed infections with *Brucella* spp. and *C. burnetii* were recordedthroughout the studied years, with 2020 having the highest rate of positive cases.

**Figure 2:**
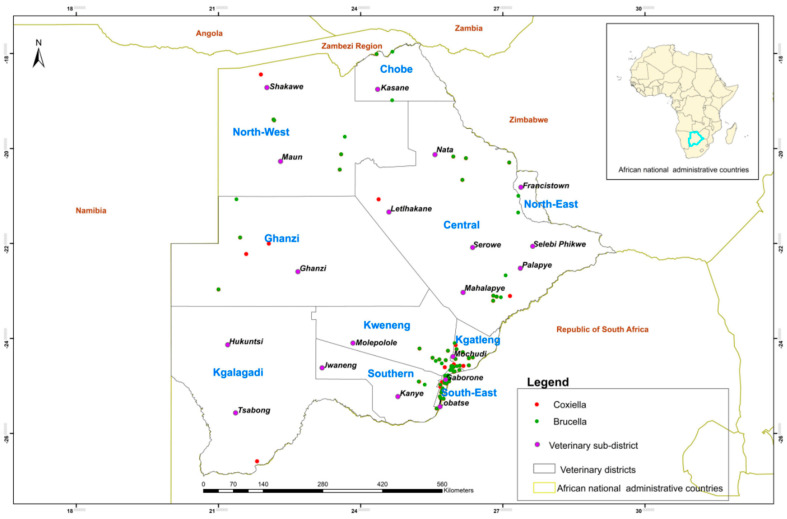
Map of Botswana showing distribution of pathogens per location.

*Brucella* spp. and *C. burnetii* were detected mainly in the Gaborone, Lobatse, Ramotswa, and Mochudi sub-districts (Figure 2).

## 4. Discussion

Accurate and rapid diagnosis of cases of abortions associated with zoonotic pathogens in livestock is important for the well-being of animals and humans, the economy of countries, and a healthy environment, as well as for the livelihoods of livestock farming communities. A multiplex testing approach was used for syndromic testing, enabling quicker identification of the causative agents of livestock abortions and enhancing disease control and management. Amongst the four zoonotic bacterial pathogens investigated, *Brucella* spp. and *C. burnetii* were detected in sheep, goats, and cattle in Botswana from abortion cases using a novel multiplex qPCR-HRM assay for the differential diagnosis of abortion infections [54]. *Brucella* spp. were the most frequently detected pathogen followed by *C. burnetii*. The findings show the potentially dominant role of *Brucella* spp. and *C. burnetii* as significant pathogens in livestock abortions in Botswana, thus highlighting the risk they might pose to the health and livelihoods of Batswana. In addition, the findings show the importance of molecular methods for differential diagnosis in detecting the presence of *Brucella* and *Coxiella* A comparison of the novel multiplex qPCR-HRM results with the well-established singleplex qPCR assays showed a perfect agreement and thus demonstrated that the multiplex qPCR-HRM assay is practical in delivering results similar to each singleplex used. In addition, the multiplex qPCR-HRM assay is easy to perform and interpret, cost-effective, and saves time in detecting abortifacient bacteria [54]. Hence, we recommend that the multiplex qPCR-HRM assay be used for the routine screening, confirmation, and molecular epidemiological surveillance of zoonotic bacteria in cattle, sheep, and goat abortions.

Most surveys found the prevalence of infection with *C. burnetii* in goats to be higher than in sheep [60,61,62,63]. Similarly, this study detected more *C. burnetii* (82.8%) in goats than in sheep (10.3%) as a single infection. This may be due to goats being more severely affected by *C. burnetii* infections than sheep, as they experience higher abortion rates and deliver weaker offspring than sheep, with some studies reporting abortion rates as high as 90% [64,65].

Even though the co-occurrence of *Brucella* spp. and *C. burnetii* is rarely reported, it is worth noting that mixed infections with these two pathogens were observed in cattle, sheep, and goats in this study, as they might have otherwise gone undetected. The co-occurrence of *Brucella* spp. and *C. burnetii* in cattle, sheep, and goats, in this study (35.5%) is significantly higher than that observed in Nigeria (1.3%) [66]. However, other studies have reported co-infection with *Brucella* spp. and *C. burnetii* in humans [67,68]. An infection with multiple pathogens can lead to more severe disease outcomes and altered transmission dynamics. *Brucella* spp. and *C. burnetii* can change host immune responses, thus likely leading to increased morbidity, mortality, and pathogen transmission during interactions between the pathogens [69]. The information obtained from this study on mixed infections can inform appropriate prevention and control strategies in Botswana to reduce the disease burden in ruminants and the potential transmission of these pathogens between livestock and humans.

*Leptospira* spp. and *L. monocytogenes* were not detected in the samples investigated, and this may be due to the low incidence of these two pathogens compared to *Brucella* spp. and *C. burnetii*. The type of samples processed for the detection of *Leptospira* spp. is also important [70]. Samples from the urinary tract (kidney and urine) are commonly used for diagnosing animal leptospirosis [71], but in this study, aborted materials were predominantly used. Most studies used milk from aborted animals, fetuses, placentas, and vaginal swabs for *Listeria* testing [72,73,74]. Future studies are nonetheless needed to actively pursue *Leptospira* testing in urinary tract samples and *Listeria* testing in dairy farms in Botswana using the multiplex qPCR-HRM assay.

Botswana employs a comprehensive approach to balance conservation efforts, livestock management, and disease control. Key strategies include veterinary cordon fences and conservation zones to separate wildlife from livestock to create disease-free areas [75,76,77,78] and reduce cross-species disease risk. Livestock movement is strictly regulated through permits and quarantine, especially from high-risk areas to reduce disease transmission, while vaccination campaigns and regular veterinary interventions support herd immunity. During outbreaks, emergency response measures, such as culling affected animals and implementing movement bans, are swiftly enacted to contain and manage the spread of disease [75,77,78].

Botswana stopped official vaccination against brucellosis in livestock in 2014 when brucellosis was under control and outbreaks appeared to decline [79]. Positive brucellosis cases at that time were as low as 3.3%. This study observed that *Brucella* positive cases rose to about 17% in 2017 and underwent a significant increase of about 38% in 2021. However, the disease situation could be more due to underreporting and some cases going unnoticed in the field. Similarly, Minas and colleagues reported an increase in the prevalence of brucellosis in animals and incidence in humans following cessation of vaccination [80]. The increase in *Brucella*-positive livestock cases in Botswana highlights the need to reintroduce vaccination programs for affected livestock species to prevent the spread of the disease to other susceptible hosts, including humans.

In examining the geographic distribution of abortifacient pathogens in domestic ruminants across Botswana, it is notable that *Brucella* spp. and *C. burnetii* were primarily detected in the sub-districts of Gaborone, Lobatse, Ramotswa, and Mochudi. The reason could be that more sample submissions came from these areas which are nearer to BNVL, where the testing of the samples was conducted. Transport could be a challenge in submitting samples to the laboratory for areas far away from the laboratory.

Importantly, we detected *Brucella* spp., and *C. burnetii* nucleic acids in raw milk samples, highlighting a public health hazard for milk handlers, workers in dairy farms, and milk consumers. The presence of *C. burnetii* and *Brucella* spp. in sheep, goat, and cow raw milk samples has been reported before [81,82,83].

As with previous studies [84,85,86,87], this study also detected *Brucella* spp. DNA in blood and serum samples from cattle, sheep, and goats.

Considering the scarcity of epidemiological data on disease occurrence in most sub-Saharan countries, the data presented in this study contribute to current scientific knowledge on the status of *Brucella* spp. and *C. burnetii* in Botswana and and the SADC region as abortifacient agents in farmed ruminants.

## 5. Conclusions

In conclusion, *Brucella* spp. and *C. burnetii* were detected in cattle, sheep, and goats in various sub-districts of Botswana. *Brucella* spp. were the most frequently detected among the four pathogens investigated. This study also confirmed the co-occurrence of *Brucella* spp. and *C. burnetii* in the domestic ruminants, being more pronounced in goats than in other ruminants. The publication of this information, coupled with public health awareness through media (audio, visual, and newspapers) and campaigns targeting local farmers, will provide the necessary information about brucellosis and coxiellosis. It will emphasize the significance of transmission between humans, livestock, and wildlife, while educating the relevant bodies regarding future recognition of these diseases, ensuring timely reporting, and facilitating appropriate treatment. This study utilized samples submitted for routine testing at the laboratory. However, country-wide surveillance, involving a larger number of samples from both intra- and inter-species studies, is needed to determine the prevalence of *Brucella* and *Coxiella* infections in Botswana’s cattle, sheep, goats, and wildlife. This would help to identify infection risks for other animals and humans, aligning with the ’One Health’ approach. We further recommendthe reintroduction of the vaccination program against brucellosis in livestock in Botswana. Also, the detection of *Brucella* spp. and *C. burnetii* should be included in the microbiological criteria for raw milk, especially when the milk is intended for direct human consumption. Promotion of milk safety measures, including pasteurization and home boiling, may contribute to reduced zoonotic spread of brucellosis and coxiellosis.

## Figures and Tables

**Table 1:** Types of specimen used in syndromic surveilance.

Specimen	*Brucella* spp./ *C. burnetii*	*Brucella* spp.	*C. burnetii*	Negative	Total
Stomach contents	20	14	5	3	42
Liver/spleen	12	2	12	17	43
Pooled foetal tissues	4	8	5	9	26
Serum	2	2	0	4	8
Whole blood	3	1	1	1	6
Placenta	4	0	1	1	6
Brain	1	0	0	0	1
Foetal brain	0	2	0	1	3
Uterus	1	1	0	0	2
Lymph node	2	0	1	0	3
Abdominal fluid	0	0	1	0	1
Abomasum	0	0	1	0	1
liver	0	0	1	1	2
Liver/kidney	1	0	0	0	1
Lung/liver/kidney	1	0	0	0	1
Lung/liver/spleen	0	1	0	0	1
Liver/spleen/kidney	0	1	0	0	1
Vaginal swab	2	0	1	0	3
Ovary tubes	1	0	0	0	1
Total	54	32	29	37	152

**Table 2:** Abortive pathogens detected in the syndromic surveillance study.

Pathogen(s) Detected	Cattle (%)	Sheep (%)	Goats (%)	Total (%)
*Brucella* spp.	9 (34.6)	3 (21.4)	20 (17.9)	32 (21.1)
*C. burnetii*	2 (7.7)	3 (21.4)	24 (21.4)	29 (19.1)
*Brucella* spp./*C. burnetii*	11 (42.3)	6 (42.8)	37 (33.0)	54 (35.5)
Negative	4 (15.4)	2 (14.3)	31 (27.7)	37 (24.3)
Total	26	14	112	152

## Data Availability

All data generated or analyzed during this study are contained within the article and Supplementary Materials.

## Supplementary Materials

The following supporting information can be downloaded at: https://www.mdpi.com/article/10.3390/microorganisms12122644/s1, Figure S1: Bland-Altman analysis. HRM versus *Brucella* spp. qPCR; Figure S2: Bland-Altman analysis. HRM versus *Coxiella burnetii*; Table S1: Livestock population per district (Source, [4]); Table S2: Samples used in molecular testing of four abortive agents in cattle, sheep, and goats; Table S3: Primer and probe sequences for the singleplex qPCR assays; Table S4: PCR cycling conditions used in the singleplex qPCR assays.
