# Supplementary material for: Molecular Testing of Zoonotic Bacteria in Cattle, Sheep, and Goat Abortion Cases in Botswana
Source: Microorganisms. 2024 Dec 20;12(12):2644. doi: 10.3390/microorganisms12122644 (PMC11728289; doi:10.3390/microorganisms12122644)
Supplement: Supplementary file 1 [file microorganisms-12-02644-s001.zip › microorganisms-3207532-supplementary.pdf]

Syndromic Testing of Zoonotic Bacteria in Cattle, Sheep, and Goat Abortion Cases in Botswana

Boitumelo M. Modise-Tlotleng [1,2], Sununguko W. Mpoloka [2], Tirumala B. K. Settypalli [3], Joseph Hyera [4], Tebogo Kgotlele [1], Kago Kumile [1], Mosarwa E. Sechele [5], Obuile O. Raboloko [5], Chandapiwa Marobela-Raborokgwe [5], Gerrit J. Viljoen [3], Giovanni Cattoli [6] and Charles E. Lamien [3,*]

[1] National Agricultural Research and Development Institute, Private Bag 0035, Gaborone, Botswana; boikensmod@gmail.com (B.M.M.-T.); tkgotlele@nardi.org.bw (T.K.); kkumile@nardi.org.bw (K.K.)

[2] Department of Biological Sciences, University of Botswana, Private Bag 00704, Gaborone, Botswana; mpoloka@ub.ac.bw

[3] Animal Production and Health Laboratory, Joint FAO/IAEA Centre of Nuclear Techniques in Food and Agriculture, Department of Nuclear Sciences and Applications, International Atomic Energy Agency, Wagramer Strasse 5, P.O. Box 100, 1400 Vienna, Austria; t.b.k.settypalli@iaea.org (T.B.K.S.); g.j.viljoen@gmail.com (G.J.V.)

[4] Botswana Vaccine Institute, Private Bag 0031, Gaborone, Botswana; jhyera@bvi.co.bw

[5] Department of Veterinary Services (DVS), Ministry of Agriculture, Private Bag 0032, Gaborone, Botswana; mesechele@gmail.com (M.E.S.); ooraboloko@gmail.com (O.O.R.); chandapiwamarobela@gmail.com (C.M.-R.)

[6] Istituto Zooprofilattico Sperimentale delle Venezie,Viale dell'Università 10, 35020 Legnaro, Italy; gcattoli@izsvenezie.it

* Correspondence: c.lamien@iaea.org; Tel.: +43-676-4649209

Supplementary Material

Table S1. Livestock population per district (Source, [4])

	Livestock population			
District	Cattle	Goats	Sheep	Total
Chobe	3,157	3,447	116	6,720
North-west	114,894	76,233	10,366	201,493
Ghanzi	70,754	25,572	7,843	104,169
Kgalagadi	45,852	43,107	25,653	114,612
Central	350,085	366,459	50,417	766,961
North-east	27,612	64,141	4,612	96,365
Kweneng	134,448	285,169	60,991	480,608
Southern	125,247	239,404	59,243	423,894
Kgatleng	59,278	94,628	20,405	174,311
South-east	3,404	30,585	3,266	37,255
Total	934,732	1,228,744	242,911	2,163,476

Table S2. Samples used in molecular testing of four abortive agents in cattle, sheep, and goats.

	ID	Year	Sample	Species	Sub district	District	No affected	No at risk	No dead
1	Bot 1725/2014	2014	FT	Caprine	Ramotswa	South East	4	40	2
2	Bot 3354/2014	2014	FT	Caprine	Gaborone	South East	No information	No information	No information
3	Bot 3065/2015	2015	Placenta	Ovine	Gaborone	South East	1	15	0
4	Bot 1024/2016	2016	Liver/spleen	Caprine	Ramotswa	South East	8	2	0
5	Bot 0807/2017	2017	Liver/spleen	Caprine	Mogobane	South East	5	35	1
6	Bot 0893/2017	2017	Liver/spleen	Caprine	Gaborone	South East	No information	No information	No information
7	Bot 1396/2017	2017	Abdominal contents	Ovine	Gaborone	South East	No sample submission form	No sample submission form	No sample submission form
8	Bot 1396_B/2017	2017	Liver/spleen						
9	Bot 2756/2017	2017	SC	Caprine	Ramotswa	South East	No information	No information	No information
10	Bot 2756_B/2017	2017	Liver/spleen						
11	Bot 2757/2017	2017	Liver/spleen	Caprine	Ramotswa	South East	No information	No information	No information
12	Bot 2884/2017	2017	Liver/spleen	Caprine	Ramotswa	South East	No information	No information	No information
13	Bot 2947/2017	2017	SC	Caprine	Tlokweng	South East	15	57	0
14	Bot 2947_B/2017	2017	Liver/spleen						
15	Bot 2992/2017	2017	Liver/spleen	Caprine	Tlokweng	South East	2	5	0
16	Bot 2992_B/2017	2017	SC						
17	Bot 3177/2017	2017	Liver/spleen	Caprine	Ramotswa	South East	No sample submission form	No sample submission form	No sample submission form
18	Bot 3223/2017	2017	Tissues	Caprine	Sebele	South East	0	207	1
19	Bot 3223_B/2017	2017	WB						
20	Bot 3531/2017	2017	Placenta	Caprine	Ramotswa	South East	No information	No information	No information
21	Bot 3616/2017	2017	Placenta	Bovine	Ramotswa	South East	1	15	0
22	Bot 0598/2019	2019	Liver/spleen	Caprine	Ramotswa	South East	1	5	0
23	Bot 1109/2020	2020	FT	Ovine	Gaborone	South East	4	100	0
24	Bot 1340/2020	2020	Placenta	Ovine	Ramotswa	South East	No information	No information	No information
25	Bot 1644/2020	2020	FT	Caprine	Mogobane	South East	1	14	0
26	Bot 1802/2020	2020	Liver/spleen	Caprine	Ramotswa	South East	1	6	0
27	Bot 1802_B/2020	2020	SC						
28	Bot 2256/2020	2020	Liver/spleen	Caprine	Mogobane	South East	12	0	12
29	Bot 2256_B/2020	2020	SC						
30	Bot 2933/2020	2020	Brain	Caprine	Lionpark	South East	19	50	Caprine-6<br>Ovine-13

31	Bot 3059/2020	2020	FT	Caprine	Gaborone	South East	18	70	0
32	Bot 3090/2020	2020	Placenta	Ovine	Gaborone	South East	No information	No information	No information
33	Bot 3340/2020	2020	Liver/spleen	Caprine	Ramotswa	South East	4	7	4
34	Bot 3391/2020	2020	WB	Caprine	Gaborone	South East	No sample submission form	No sample submission form	No sample submission form
35	Bot 3392/2020	2020	WB	Ovine	Gaborone	South East	No sample submission form	No sample submission form	No sample submission form
36	Bot 808/2021	2021	V. swab	Caprine	Ramotswa	South East	2	40	0
37	Bot 808_B/2021	2021	WB						
38	Bot 808_C/2021	2021	SC						
39	Bot 1274/2021	2021	SC	Caprine	Ramotswa	South East	6	40	0
40	Bot 1274_B/2021	2021	FT						
41	Bot 1889/2021	2021	Liver/spleen	Caprine	Tlokweng	South East	1	44	3
42	Bot 1889_B/2021	2021	SC						
43	Bot 1976/2021	2021	SC	Bovine	Gaborone	South East	No information	No information	No information
44	Bot 2138/2021	2021	SC	Caprine	Gaborone	South East	1	35	1
45	Bot 2138_B/2021	2021	FT						
46	Bot 2332/2014	2014	SC	Caprine	Lobatse	Southern	2	40	0
47	Bot 2881/2016	2016	SC	Bovine	Goodhope	Southern	No information	No information	No information
48	Bot 3224/2017	2017	FT	Caprine	Kanye	Southern	No information	No information	No information
49	Bot 1528/2019	2019	Cultured tissue-DNA	Bovine	Lobatse	Southern	1	0	0
50	Bot 1530/2019	2019	Cultured LN-DNA	Bovine	Lobatse	Southern	2	900	0
51	Bot 1530_B/2019	2019	ovary tubes						
52	Bot 3176/2019	2019	SC	Bovine	Lobatse	Southern	No information	No information	No information
53	Bot 3303/2019	2019	Liver/kidney	Bovine	Lobatse	Southern	1	900	0
54	Bot 4258/2019	2019	Liver/spleen	Bovine	Lobatse	Southern	1	400	0
55	Bot 4326/2019	2019	Liver/spleen	Bovine	Lobatse	Southern	4	0	0
56	Bot 0686/2020	2020	Liver/spleen	Bovine	Lobatse	Southern	No information	No information	No information
57	Bot 1571/2020	2020	LN 15057957	Bovine	Lobatse	Southern	No information	No information	No information
58	Bot 1571_B/2020	2020	Serum						
59	Bot 1571_C/2020	2020	LN 15944657						
60	Bot 2859/2020	2020	Serum	Bovine	Lobatse	Southern	No sample submission form	No sample submission form	No sample submission form
61	Bot 1909/2020	2020	SC	Ovine	Lobatse	Southern	No information	No information	No information
62	Bot 3671/2020	2020	Serum	Bovine	Lobatse	Southern	43	87	0
63	Bot 3676/2020	2020	Serum	Bovine	Lobatse	Southern	43	53	0

64	Bot 3677/2020	2020	Serum	Bovine	Lobatse	Southern	68	100	0
65	Bot 0033/2021	2021	FT	Bovine	Lobatse	Southern	1	900	0
66	Bot 0677/2021	2021	Serum	Bovine	Kanye	Southern	0	0	0
67	Bot 0711/2021	2021	Serum	Bovine	Lobatse	Southern	0	0	0
68	Bot 1064/2021	2021	FT	Bovine	Lobatse	Southern	0	0	0
69	Bot 1956/2021	2021	Liver/spleen	Ovine	Kanye	Southern	5	1007	1
70	Bot 1956_B/2021	2021	SC						
71	Bot 2082/2021	2021	SC	Caprine	Moshana	Southern	15	50	0
72	Bot 1162/2017	2017	Liver/spleen	Caprine	Mochudi	Kgatleng	1	0	0
73	Bot 3457/2017	2017	FT	Caprine	Mochudi	Kgatleng	No information	No information	No information
74	Bot 1318/2018	2018	Liver/spleen	Caprine	Bokaa	Kgatleng	2	100	0
75	Bot 1327/2018	2018	FT	Caprine	Bokaa	Kgatleng	3	19	1
76	Bot 0846/2019	2019	SC	Ovine	Malotwane	Kgatleng	No information	No information	No information
77	Bot 1228/2019	2019	SC	Ovine	Malotwane	Kgatleng	No information	No information	No information
78	Bot 1359/2019	2019	Liver/spleen	Caprine	Dikgonnye	Kgatleng	1	0	1
79	Bot 1792/2020	2020	SC	Caprine	Malotwane	Kgatleng	2	32	0
80	Bot 1792_B/2020	2020	Liver/spleen						
81	Bot 2132/2020	2020	Liver/spleen	Caprine	Mochudi	Kgatleng	7	148	0
82	Bot 2175/2020	2020	SC	Caprine	Mochudi	Kgatleng	No information	No information	No information
83	Bot 2262/2020	2020	SC	Caprine	Mochudi	Kgatleng	No information	200	20
84	Bot 2262_B/2020	2020	FT						
85	Bot 2833/2020	2020	FT	Caprine	Mochudi	Kgatleng	No information	No information	No information
86	Bot 2833_B/2020	2020	SC						
87	Bot 1659/2021	2021	Liver/spleen	Caprine	Mochudi	Kgatleng	70	70	5
88	Bot 1659_B/2021	2021	SC						
89	Bot 2513/2021	2021	FT	Caprine	Oodi	Kgatleng	30	10	0
90	Bot 2513_B/2021	2021	SC						
91	Bot 4047/2017	2017	Liver	Caprine	Molepolole	Kweneng	1	38	0
92	Bot 1820/2019	2019	Placenta	Caprine	Molepolole	Kweneng	0	0	4
93	Bot 3040/2019	2019	Liver/spleen	Caprine	Kopong	Kweneng	14	47	0
94	Bot 1883/2020	2020	SC	Caprine	Kopong	Kweneng	5	45	0
95	Bot 2395/2020	2020	SC	Caprine	Ramakgatlanyane	Kweneng	1	14	0
96	Bot 2428/2020	2020	FT	Caprine	Lentsweletau	Kweneng	6	40	0
97	Bot 2428_B/2020	2020	SC						

98	Bot 819/2021	2021	SC	Caprine	Sandveld	Kweneng	35	175	1
99	Bot 819_B/2021	2021	Liver/spleen						
100	Bot 902/2021	2021	Brain	Caprine	Molepolole	Kweneng	4	19	2
101	Bot 902_B/2021	2021	FT						
102	Bot 952/2021	2021	SC	Caprine	Molepolole	Kweneng	No sample submission form	No sample submission form	No sample submission form
103	Bot 952_B/2021	2021	Liver/spleen						
104	Bot 1368/2021	2021	SC	Caprine	Letlhakeng	Kweneng	3	46	0
105	Bot 2041/2021	2021	SC	Caprine	Molepolole	Kweneng	1	70	0
106	Bot 2041_B/2021	2021	FT						
107	Bot 2590/2021	2021	Uterus	Caprine	Mogoditshane	Kweneng	No information	No information	No information
108	Bot 1309/2017	2017	Liver/spleen	Caprine	Nata	Central	No information	No information	No information
109	Bot 1310/2017	2017	Liver/spleen	Caprine	Sowa	Central	7	200	3
110	Bot 1315/2017	2017	Liver/spleen	Caprine	Nata	Central	No information	No information	No information
111	Bot 2928/2017	2017	Liver/spleen	Caprine	Mahalapye East	Central	14	265	0
112	Bot 2929/2017	2017	Liver	Caprine	Mahalapye East	Central	14	265	0
113	Bot 2930/2017	2017	Liver/spleen	Caprine	Mahalapye East	Central	1	6	0
114	Bot 3907/2017	2017	Lung/liver/kidney	Caprine	Mahalapye	Central	No information	No information	No information
115	Bot 4122/2017	2017	V. swab 1554-DNA	Caprine	Rakops Hardveld	Central	20	100	0
116	Bot 4122_B/2017	2017	V. swab 1553 - DNA						
117	Bot 2138/2020	2020	Liver/spleen	Caprine	Morale East	Central	7	140	0
118	Bot 2140/2020	2020	Liver/spleen	Caprine	Shakwe	Central	1	25	0
119	Bot 2419/2020	2020	FT	Caprine	Mapose	Central	0	5	4
120	Bot 2883/2020	2020	SC	Caprine	Tonota East	Central	4	33	0
121	Bot 0439/2021	2021	WB	Caprine	Tonota East	Central	2	60	0
122	Bot 0523/2021	2021	FT	Caprine	Mahalapye East	Central	1	29	3
123	Bot 654/2021	2021	Serum	Bovine	Nata	Central	No sample submission form	No sample submission form	No sample submission form
124	Bot 1048/2021	2021	SC	Caprine	Nkange	Central	58	30	0
125	Bot 1048_B/2021	2021	FT						
126	Bot 1175/2021	2021	SC	Caprine	Nata	Central	No sample submission form	No sample submission form	No sample submission form
127	Bot 1623/2021	2021	SC	Caprine	Taupye	Central	0	40	0
128	Bot 1623_B/2021	2021	Liver/spleen						
129	Bot 2056/2021	2021	FB	Ovine	Radisele	Central	No information	No information	No information

130	Bot 2745/2021	2021	FB	Caprine	Tonota West	Central	4	11	4
131	Bot 2745_B/2021	2021	SC						
132	Bot 2745_C/2021	2021	Liver/spleen						
133	Bot 0667/2015	2015	FT	Caprine	Nata	North East	0	60	0
134	Bot 0667_B/2015	2015	WB						
135	Bot 0596/2017	2017	Liver/spleen	Caprine	Parakarungu	Chobe	10	23	2
136	Bot 1894/2014	2014	SC	Bovine	Mabele	Chobe	2	25	0
137	Bot 1100/2019	2019	Liver/spleen	Ovine	Pandamatenga	Chobe	1	30	0
138	Bot 2562_B/2021	2021	Liver/spleen/kidney	Bovine	Kavimba	Chobe	0	36	6
139	Bot 1920/2020	2020	SC	Bovine	Hainaveld	North-West	2	0	0
140	Bot 2112/2020	2020	FT	Caprine	Gumare	North-West	3	22	0
141	Bot 2116/2020	2020	Liver/spleen	Caprine	Chanoga	North-West	12	53	0
142	Bot 2116_B/2020	2020	SC						
143	Bot 2556/2020	2020	SC	Caprine	Gumare	North-West	8	50	0
144	Bot 2556_B/2020	2020	Liver/spleen						
145	Bot 3175/2020	2020	Liver/spleen	Caprine	Shakawe	North-West	2	105	2
146	Bot 0735/2021	2021	FT	Caprine	Shakawe	North-West	No information	No information	No information
147	Bot 1882/2021	2021	Liver/spleen/lung	Caprine	Shorobe	North-West	11	15	11
148	Bot 2367/2021	2021	SC	Caprine	Gumare	North-West	4	15	11
149	Bot 1676/2019	2019	SC	Caprine	Ghanzi South	Ghanzi	0	26	0
150	Bot 2046/2020	2020	Uterus	Caprine	Ghanzi South	Ghanzi	1	15	1
151	Bot 2330/2020	2020	Liver/spleen	Caprine	New Xade	Ghanzi	0	0	5
152	Bot 3345/2020	2020	Abomasum	Caprine	Middlepits	Kgalagadi	30	200	30

FT: pooled foetal tissues FB: Foetal brain SC: Stomach contents WB: Whole blood V: Vaginal LN: Lymph node

Table S3. Primer and probe sequences for the singleplex qPCR assays

Infectious agent	Target gene	Primer and probe sequences and concentrations	Concentrations
Brucella spp.	*IS711*	F:5′-GCTTGAAGCTTGCGGACAGT-3′	300 nM
		R:5′-GGCCTACCGCTGCGAAT-3′	300 nM
		P: FAM-5′-AAGCCAACACCCGGCCATTATGGT-3′-BHQ1	200 nM
C. burnetii	*IS1111*	F: 5′-CCGATCATTTGGGCGCT-3′	1600 nM
		R: 5′-CGGCGGTGTTTAGGC-3′	800 nM
		P: FAM-5′-TTAACACGCCAAGAAACGTATCGCTGTG-3′-BHQ1	200 nM
Leptospira spp.	*lipL32*	F: 5′-CCCTAIGGATCTGTRATCAACTA-3'	900 nM
		R: 5′-GAACTCCCATTTCAGCGATT-3'	900 nM
		P: FAM-5′-AAAGCCAGGACAAGCGCCG-3′-BHQ1	250 nM
L. monocytogenes	*ssrA*	F: 5′-CGTGCATCGCCCATGTGC-3'	300 nM
		R: 5′-ATCTACGAGCGTAGTCAC-3'	300 nM

Table S4. PCR cycling conditions used in the singleplex qPCR assays

Infectious agent	Cycling conditions
Brucella spp.	95˚C - 5 min,<br>45 cycles: 95˚C - 15 s, 60˚C – 60 s
C. burnetii	95˚C - 10 min,<br>40 cycles: 95˚C - 15 s, 60˚C – 60 s
Leptospira spp.	50˚C – 10 min,<br>95˚C – 20 s,<br>45cycles: 95˚C - 3 s, 60˚C – 30 s
L. monocytogenes	95°C - 5 min,<br>45 cycles: 95°C - 15 s, 60°C - 60 s,<br>Melting:<br>95°C - 60 s,<br>65 °C - 60 s,<br>65°C - 95°C in 0.5°C/0.05s increments

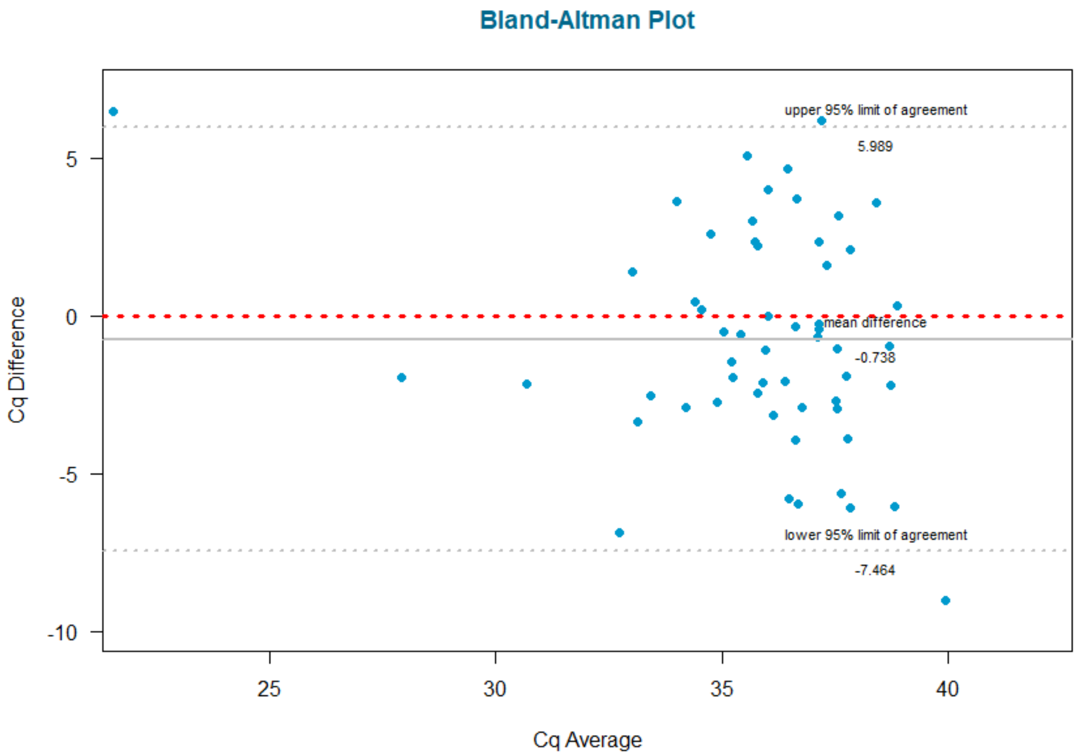

Figure S1. Bland-Altman analysis. HRM versus *Brucella* spp qPCR

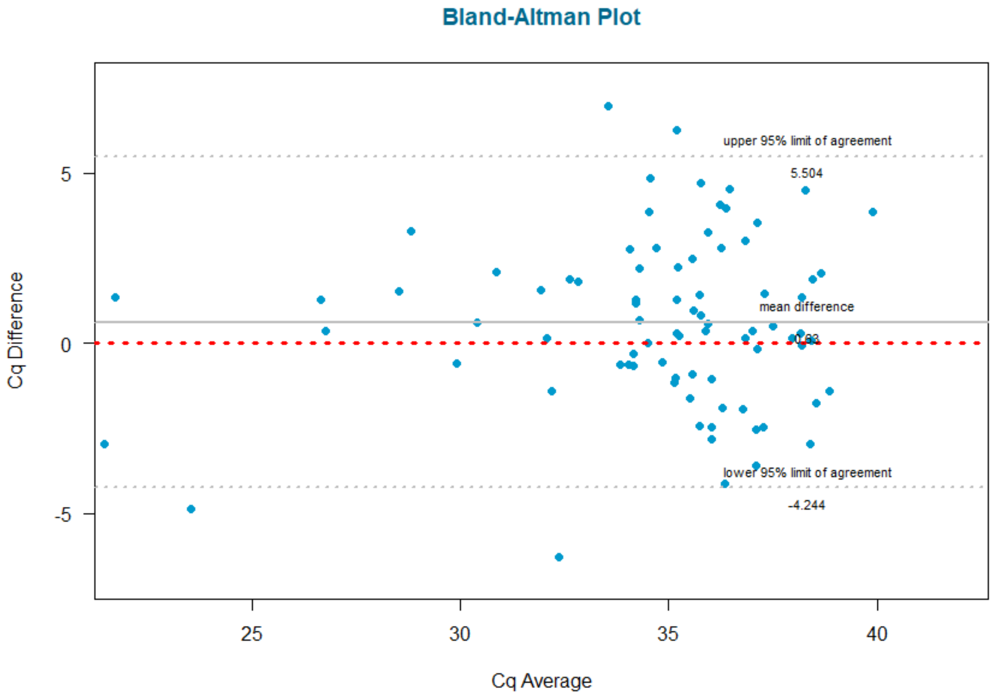

Figure S2. Bland-Altman analysis. HRM versus *Coxiella burnetii*
